# Characterization of Extraintestinal Pathogenic *Escherichia coli* from Human Clinical and Poultry Samples

**DOI:** 10.3390/microorganisms13112603

**Published:** 2025-11-15

**Authors:** Manita Guragain, Lori Bagi, Yanhong Liu, Joseph M. Bosilevac

**Affiliations:** 1 Characterization and Intervention for Foodborne Pathogens Research Unit, Eastern Regional Research Center, U.S. Department of Agriculture, Agricultural Research Services, Wyndmoor, PA 19038, USA; lori.bagi@usda.gov (L.B.); yanhong.liu@usda.gov (Y.L.); 2 Meat Safety and Quality Research Unit, U.S. Meat Animal Research Center, U.S. Department of Agriculture, Agricultural Research Services, Clay Center, NE 68933, USA; mick.bosilevac@usda.gov

**Keywords:** extraintestinal pathogenic *E. coli*, poultry, antimicrobial resistance, host-cell adherence

## Abstract

Extraintestinal Pathogenic *Escherichia coli* (ExPEC) are leading causes of adult bacteremia, neonatal meningitis, pneumonia, and the vast majority of urinary tract infections. ExPEC are present in food, mostly in poultry products. Despite the high burden of ExPEC on human health, their source and impact on food safety are largely unknown. Food-isolated ExPEC carry an abundance of virulence and antibiotic resistance genes and are suggested to have zoonotic potential. Here, ExPEC were characterized for sanitizer tolerance, biofilm formation, antibiotic susceptibility, and adherence to Caco-2 cells (a human intestinal epithelial-derived cell line). The ExPEC used were isolated from poultry meat and from archived collections of poultry and human clinical samples. Overall, low frequency of sanitizer tolerance (2.4%) and biofilm formation (6%) were observed under the test conditions used. The frequency of antibiotic susceptibility was significantly higher (*p* < 0.05) among human clinical ExPEC (55%) compared to poultry-isolated ExPEC (27.9%). Overall, 62.8% of poultry ExPEC showed adherence to human intestinal epithelia at a level comparable to that of the control enteric pathogen *E. coli* O157:H7. In summary, the results of this work suggest that the human gastrointestinal tract could serve as a reservoir of high-risk poultry ExPEC if consumed, and, hence, it is a potential source of extraintestinal infections.

## 1. Introduction

Extraintestinal pathogenic *Escherichia coli* (ExPEC) are a diverse group of *E. coli* infecting bodily sites other than the gastrointestinal (GI) tract and cause a wide range of human infections (as reviewed in [1]). Subtypes of ExPEC are defined by the specific diseases they cause: Sepsis-causing *E. coli* (SEPEC), Neonatal meningitis-causing *E. coli* (NMEC), and Uropathogenic *E. coli* (UPEC) [2]. Systematic reviews of 210 studies between 2007 and 2018 identified the incidence rate of *E. coli* bacteremia to be 48 per 100,000 persons, with higher incidence among the elderly [3]. NMEC is the most common Gram-negative bacterium responsible for meningitis in newborns accounting for 13–33% of cases which are associated with high mortality [4,5,6]. UPEC are the major bacterial causative agent of urinary tract infections (UTIs), of which the annual global burden during 2019 was 404.61 million [7], (as reviewed in [8]). In the United States, 75–95% of all uncomplicated kidney and bladder infections are caused by UPEC. Additionally, UTIs are a primary source of *E. coli* bacteremia [3]. Despite this high burden of ExPEC on human health, their source and transmission are not well understood.

A growing body of research shows the presence of ExPEC in food, mostly in poultry [9,10,11,12,13,14,15,16,17,18,19]. A large-scale genomics study reported an abundance of virulence and antibiotic resistance genes in ExPEC from food sources, with almost 10% of the food isolates predicted to present a medium–high risk to humans [20]. Sequence types similar to ExPEC isolates from suspected cases of UTI were found in retail chicken and turkey [21]. *E. coli* sequence type ST131, which is the most prevalent and multidrug resistant UPEC, is established in poultry populations around the world [12]. Poultry-derived ExPEC strains of ST95 are suggested to have zoonotic potential [22]. ExPEC of poultry origin were also shown to be clonally related to human UPEC and exhibited virulence in animal models of human UTI and neonatal meningitis [23,24,25,26].

Foodborne illness continues to be a significant public health threat despite continuous prevention and control measures. The Centers for Disease Control and Prevention (CDC) reported 28 outbreaks linked to contaminated food during 2021, with major food sources being chicken and leafy greens [27]. Historically, foodborne pathogens are associated with enteric symptoms, often acute, resulting from consumption of contaminated foods [27]. In addition to classic enteric foodborne pathogens, understanding any emerging foodborne pathogen and its impact on food safety is warranted for comprehensive food safety. The presence of non-diarrheagenic pathogenic *E. coli* like genotoxic *E. coli* and ExPEC in food has been reported [28,29,30]. Therefore, this study aims to characterize ExPEC isolated from poultry meats and human clinical samples for antibiotic susceptibility, sanitizer tolerance, biofilm formation, and host cell attachment to better understand the risk of poultry-borne ExPEC on human infections.

## 2. Materials and Methods

Study design. Human clinical ExPEC isolates (N = 40) from previous studies were included in this study. Among 40 human clinical ExPEC, 30 were from UTI cases, and 5 were from neonatal meningitis cases. Metadata for the remaining 5 archived human isolates were not available. Forty-three ExPEC isolates from poultry meat sources were included in the study. Among these, eight were previously isolated from retail chicken meat [19]. Twelve out of fifty-four *E. coli* from the *E. coli* reference center at Penn State University were confirmed to be ExPEC using PCR as described below and included in the study. Additionally, 103 poultry meat enrichments (chicken, n = 88; turkey, n = 15) from three different geographical regions of the United States (Eastern, n = 24; Western, n = 29; and Central, n = 50) were screened using PCR, and 23 ExPEC isolates were recovered and included in the study.

Screening of ExPEC isolates. Template DNA was prepared by mixing overnight cultures of ExPEC growing at 37 °C in Luria-Bertani (LB) broth (Fisher Scientific, Pittsburg, PA, USA) with Bax lysis buffer (Hygiena, Camarillo, CA, USA) following the manufacturer’s recommendations. To screen for ExPEC, two multiplex PCR using primers targeting virulence gene markers (*afa/dra*, *iutA*¸ *kpsMT II*, *papA* or *papC*, and *sfa/foc*) were utilized as previously described [31]. Markers *papA* and *papC* were amplified separately but analyzed as a combined single marker. Amplicons were resolved on 2% agarose gels and visualized using Gel-Red (Thomas Scientific, Swedesboro, NJ, USA).

Antibiotic susceptibility assay. Antibiotic susceptibility was tested using the Kirby Bauer disc diffusion method. Briefly, isolated colonies of *E. coli* on Brain Heart Infusion agar (BHIA; Becton Dickinson, Sparks, MD, USA) were resuspended in sterile saline to match the turbidity of a 0.5 MacFarland standard (Thermo Fisher Scientific, Waltham, MA, USA). The cell suspension was swabbed over the surface of Muller Hinton Agar (MHA, Millipore Sigma, Burlington, MA, USA), and individual antibiotic discs (Ampicillin 10 µg, Cefepime 30 µg, Ceftazidime 30 µg, cephalothin 30 µg, chloramphenicol 30 µg, ciprofloxacin 5 µg, doxycycline 30 µg, ertapenem 10 µg, fosfomycin 200 µg, gentamycin 10 µg, kanamycin 30 µg, nalidixic acid µg, nitrofurantoin 100 µg, piperacillin 100 µg, piperacillin-tazobactam, streptomycin 10 µg, sulfamethoxazole-trimethoprim (BD Sensi-disc, Becton Dickinson, Heidelberg, Germany)) were dispensed on the agar surface followed by incubation at 35 °C for 18 h. The zone of inhibition was measured and interpreted according to CLSI disc zone interpretation standards [32]. The difference in frequency of antibiotic/antimicrobial resistance (AMR) was compared using Fisher’s Exact test.

Sanitizer tolerance. Isolated colonies of *E. coli* in BHI agar were inoculated into Luria-Bertani broth with no added salt (LB-NS; Becton Dickinson, Sparks, MD, USA) and incubated at 37 °C for 18 h. Overnight cultures were adjusted to an optical density (600 nm) of 0.6 to 0.7 by addition of LB-NS to yield a culture of approximately 1 × 10^8^ CFU/mL. Two hundred microliters of adjusted cultures were mixed with equal volume of 600 ppm quaternary ammonium compound solution (QAC, Vanquish, Total Solutions, Milwaukee, WI, USA), followed by a 1 min incubation at room temperature. For the non-treated control, an equal volume of sterile, deionized water was used. At the end of treatment time, 100 μL of treated cells were transferred to 900 μL D/E neutralization broth (DE; BBL, Difco, Sparks, MD, USA) and held at room temperature for 1 h. Neutralized samples were serially diluted in sterile phosphate-buffered saline (PBS; Sigma–Aldrich, St. Louis, MO, USA). For primary screening, 8 μL of appropriate dilutions were plated on nutrient agar plate (NA; Oxoid, Hampshire, UK) using a modified 6 × 6 drop plate method (Chen et al., 2003 [33]). Decreased QAC sensitivity during initial screening was confirmed by plating 100 μL of sample on to a plate of nutrient agar. Plates were incubated at 37 °C for 18 h. Colonies were counted under a Stemi 508 microscope (Zeiss, Oberkochen, Germany), CFU/mL, and log reduction in viable cells was calculated and compared against the untreated samples. Less than 3 log reductions in CFU were considered resistant, whereas more than 5 log reductions in CFU were considered sensitive following guidelines for sanitizers used on hard surfaces [34]. For each isolate, two or more rounds of experiments, each including three replicates, were performed.

Biofilm formation. Biofilm forming potential was evaluated using a microtiter plate assay. Isolated colonies of *E. coli* on BHI agar were inoculated in LB-NS and incubated at 37 °C for 18 h with shaking (150 rpm). Overnight culture was diluted 1:100 in LB-NS, and 100 µL was placed in three wells of a 96-well Corning Costar microtiter plate (Fisher Scientific, Waltham, MA, USA). Previously identified strong biofilm−forming *E. coli* O157:H7 (strain 43894OR 22/30 (Uhlich et al., 2001 [35])) was used a positive control, and sterile BHI broth was used as a negative control. Plates were incubated at 30 °C for 48 h inside a humidity chamber. After incubation, loosely adhered cells were removed by washing two times with 200 μL sterile water. Attached biofilm mass was stained with 100 μL of 0.1% crystal violet (CV; Sigma-Aldrich, St. Louis, MO, USA) for 45 min at room temperature. The unbound CV was removed by washing two times with sterile water. The CV bound to biofilm was extracted with 200 μL of absolute ethanol (DLI, King of Prussia, PA, USA), and absorbance at 595 nm was measured using Safire microplate reader (Tecan, Männedorf, Switzerland). Each experiment was repeated in at least two times. Mean absorbance and standard deviation for each sample were calculated, and the difference in biofilm mass was estimated using one-way ANOVA in Prism (version 10.0.1, GraphPad Software LLC, La Jolla, CA, USA). In each batch of biofilm assays, the ExPEC were compared to the positive control and ranked for biofilm biomass, with the positive control ranked highest at 3 and negative control at 0.

Host cell adhesion assay. Caco-2 cells (ATCC HTB-37) were cultured in Dulbecco’s Modified Eagle Medium (DMEM) (Fisher Scientific) supplemented with 10% heat-inactivated fetal bovine serum (FBS) (Fisher Scientific), 1% GlutaMAX (Fisher Scientific), 1% non-essential amino acids (Fisher Scientific), and 1% Penicillin-Streptomycin (Fisher Scientific). The cells were passaged at a 1:5 ratio according to the handling procedure for Caco-2 cells from ATCC. For cell adhesion assays, Caco-2 cells were seeded at 1.5 × 10^5^ cells/mL in 24-well plates with antibiotic-free medium and incubated at 37 °C, 5% CO_2_ for ~48 h until 85–95% confluency. The cell adhesion assays were performed as previously described [36] with the following modifications: *E. coli* O157:H7 (positive control) and other *E. coli* strains were cultured overnight in Luria-Bertani (LB) broth at 37 °C with shaking (180 rpm), normalized by OD_600_, and diluted in 1X Phosphate Buffered Saline (PBS, Fisher Scientific) to a multiplicity of infection (MOI) of 100:1. LB broth (1 mL) was used as a negative control. Caco-2 cell monolayers were washed with 1X PBS (pH 7.4), overlaid with antibiotic-free medium, and infected with 1 mL bacterial suspension for 3 h at 37 °C, 5% CO_2_. Non-adherent bacterial cells were removed by washing three times with 1X PBS. Caco-2 cells were lysed with 1 mL of 0.1% Triton X-100 for 10 min at room temperature, and lysates were collected by gentle scraping using a cell scraper. Serial 10-fold dilutions were prepared in LB broth, and 10 µL of each dilution were plated on LB agar in triplicate. After overnight incubation at 37 °C, colonies were counted at each dilution and averaged. The percentage of bacterial cell adhesion was the ratio between the number of adhering bacterial cells and the initial infecting bacterial cells, which was calculated as follows: CFU/mL = 100 * dilution fold * colony counts. All values represented were the average of the three experiments with standard deviations.

## 3. Results and Discussion

### 3.1. ExPEC Prevalence in Poultry Samples

ExPEC reservoirs and transmission are poorly understood. The presence of ExPEC in food samples has been reported in previous studies. During a survey of vegetable, fruit, and raw meat items between 1999 and 2000, only turkey samples tested positive for ExPEC [37]. A high prevalence of ExPEC strains was reported in poultry products during a survey of 1648 retail food items during 2001–2003 in the Midwest of the US. In our study, approximately 36% (37/104) of poultry meat enrichment samples tested positive for ExPEC. From each positive sample, five to six E. coli were isolated, and twenty ExPEC-positive samples (chicken, 18/89; turkey, 2/15) yielded 23 ExPEC isolates (chicken, 20; turkey, 3). Hence, the prevalence of ExPEC in these poultry meat samples was determined to be 19% (20/104). Similarly, 22% (12/54) of poultry *E. coli* isolates from the *E. coli* Reference Center were identified as ExPEC. Among these, 11 were chicken isolates (n = 53), and 1 was a turkey isolate (n = 1). In keeping with our observation, the prevalence of ExPEC in raw-cut chicken parts was estimated to be 38% (n = 55), with approximately 21% (n = 110) of isolates from raw-cut chicken parts identified to be ExPEC [38]. Therefore, findings from this current and previous research show poultry can serve as a potential reservoir of ExPEC.

### 3.2. Antibiotic Susceptibility Among ExPEC

**Figure 1:**
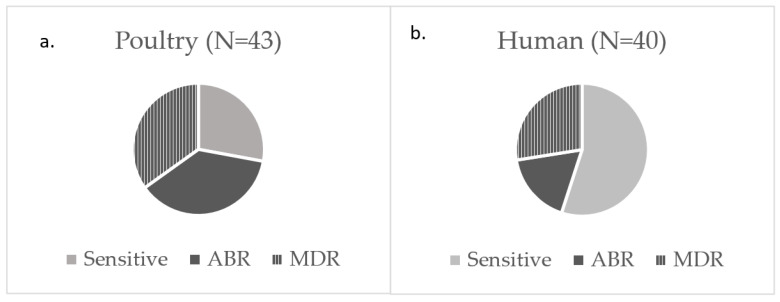

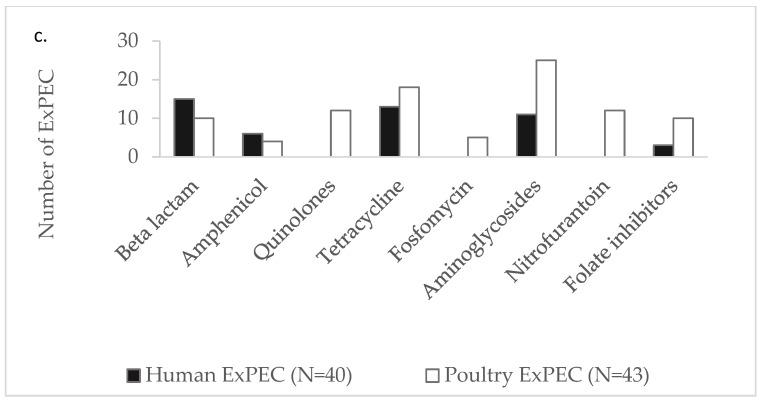
Frequency of antibiotic resistance. (**a**) Poultry ExPEC. (**b**) Human ExPEC. Light grey: sensitive; dark grey: antibiotic resistant; striped grey: multidrug resistant. (**c**) Against different antibiotic class. Black: human ExPEC; white: poultry ExPEC.

Antibiotics are routinely recommended for the treatment of ExPEC infections. A global survey between May 2019 and July 2019 reported that 36% of E. coli from bloodstream infection and 43.3% from urinary tract infections were resistant to third-generation cephalosporin and fluoroquinolones, respectively [39]. Drug-resistant ExPEC is a major cause of treatment failure posing a major health threat [40]. When the antibiotic susceptibility of ExPEC to 18 antibiotics belonging to eight different classes (beta-lactam, amphenicol, quinolones, tetracycline, fosfomycin, aminoglycoside, nitrofurantoin, and folate inhibitors) was evaluated, 59% (49/83) showed an antibiotic-resistant (ABR) phenotype (Table 1, Figure 1a,b). Fifty-three percent (26/49) of the ABR isolates displayed resistance to three or more antibiotic classes, meeting the definition of multidrug resistant (MDR) (Figure 1c). The frequency of ABR was significantly (*p* < 0.05) higher among poultry ExPEC (72%, 31/43) compared to human ExPEC (45%, 18/40) (Figure 1a,b); however, it should be noted that ABR was variable among poultry isolates coming from different sample sets. For instance, all (11/11) of the ExPEC sourced to chicken from Mexico were MDR, and seven of the nine ExPEC sourced from retail chicken meat were ABR with two of those MDR, while half (12/23) of those isolated here were ABR, with three being MDR. A higher association of ABR among poultry isolates compared to other sample sources has also been reported in a recent study of 279 ExPEC [41]. Restrospective cross-sectional studies of clinical UPEC strains from the United States have reported significant variations in ABR and MDR profiles across different variables like geographical regions (Hine et al., 2015 [42]), patient demographics, and severity of UTI (CDC, (https://arpsp.cdc.gov/profile/antibiotic-resistance/mdr-ecoli, accessed on 30 July 2025). In the current study, overall ABR frequency among ExPEC from the US was highest in the Southeast region and lowest in the Plains region. The comparison of ABR profiles between poultry and human clinical isolates across geographical regions was not attempted because of a lack of comparable sample size and corresponding metadata. Further, due to a lack of metadata regarding antibiotic use, whether different selective pressure induced by use of antibiotics among individual sample sets contributed to the difference in ABR frequency could not be assessed in our study. The highest frequency of ABR was observed against aminoglycosides (43%, 36/84), tetracyclines (37%, 31/84), beta-lactams (30%, 25/84), folate inhibitors (15.5%, 13/84), quinolones and nitrofurantoin (14.3%, 12–13/84), and amphenicol (12%, 10/84) classes (Figure 1c). No resistance was observed against three beta-lactams, namely cefepime, ertapenem, and piperacillin-tazobactam. Additionally, none of the human isolates were resistant to ciprofloxacin, fosfomycin, nalidixic acid, and nitrofurantoin (Figure 1b). One of the poultry isolates was resistant to all classes of antibiotics. Therefore, the resistance of poultry ExPEC to commonly recommended antibiotics for ExPEC infections highlights the potential for poultry to act as an important reservoir for difficult-to-treat ExPEC.

### 3.3. Sanitizer Tolerance of ExPEC

Non-thermal (high pressure processing, gamma radiation, UV-C radiation, and LED array) as well as thermal processing treatments are reported to inactivate ExPEC levels in ground chicken meat, chicken skin, and chicken purge [51,52]. Sanitation of meat processing surfaces is critical to reduce any bacterial load and prevent cross-contamination of food products. Quaternary ammonium compounds (QAC) are routinely used for disinfection of various surfaces in food processing as well as hospital environments. Further, ExPEC isolates from poultry farms have been reported to carry genes associated with resistance to disinfectants [53]. Therefore, the sensitivity of ExPEC to 300 ppm QAC was tested. Most of the ExPEC isolates (97.6%, 81/83) were reduced by 5 log CFU or more after a 1 min treatment with 300 ppm QAC and were considered sensitive (Table 1). Despite higher ABR frequency among poultry ExPEC, QAC sensitivity was not different between human and poultry ExPEC (Fisher’s exact test, *p* > 0.05). This is contrary to the positive association reported between ABR and sanitizer tolerance which has been suggested to be mediated by common efflux pumps, co-transmission of resistance genes, and co-localization of antibiotic and disinfectant resistance genes on the same plasmid or mobile genetic elements [54,55,56,57,58,59]. The independence of ABR and sanitizer tolerance in the current study suggests QAC-tolerant mechanism(s) are independent from ABR determinants and/or a possible difference in exposure history which would otherwise potentially lead to selection of co-resistant strains in the presence of relevant stress. One ExPEC from a human clinical sample was QAC-resistant based on a reduction of less than 3 log CFU. Intermediate resistance (3–5 log reductions in CFU count) was observed for the other two ExPEC isolates (poultry, n = 1 and human, n = 1), hence considered intermediately resistant. The intermediately resistant poultry isolate was ABR, illustrating a potential compound risk of poultry ExPEC on human health.

### 3.4. Biofilm Formation by ExPEC

Biofilms are mechanisms for bacterial adaptation in various environments and are known to protect the cells from harsh environmental conditions like antibiotics, sanitizers, or environmental stresses (as reviewed in [60,61,62]). Biofilms may also have variable roles in different stages of host infection [63,64]. Therefore, the biofilm formation by ExPEC strains in LB-NS medium was evaluated. Most ExPEC (79/83) formed little to no biofilm mass (Table 1). One poultry isolate formed strong biofilm comparable to the hyper biofilm-forming positive control strain used in this study. Two multidrug-resistant poultry ExPEC formed moderate biofilm. Positive correlations of biofilm with certain phylogroups, as well as ABR profiles among poultry ExPEC, have been reported previously [65]. In contrast to the results here, a majority (61%) of clinical ExPEC strains isolated from blood, respiratory samples, and urine were reported to form moderate-to-strong biofilm in minimal media [66]. Further, poultry ExPEC were observed to form higher biofilm mass compared to clinical UPEC strains [65]. The discrepancies in ExPEC biofilm phenotypes found here compared to previous reports may be due to differences in genetic background and/or the culture media used, as was suggested by [67].

### 3.5. Adherence of Poultry ExPEC to Human Epithelial Cells

**Figure 2:**
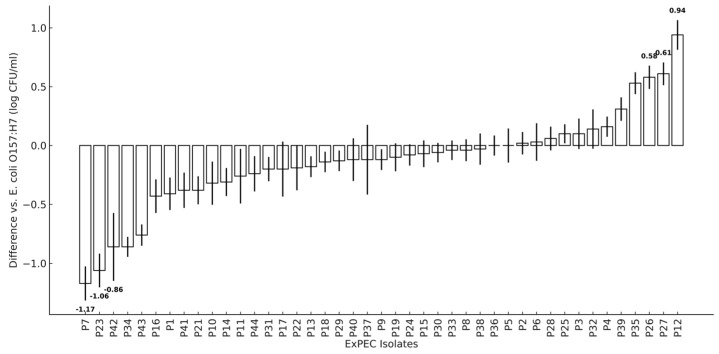
Caco-2 cell adhesion differences (log CFU/mL) of 43 ExPEC isolates compared to *E. coli* O157:H7, sorted by values. Standard deviations are indicated by black lines. The three isolates (P12, P27, P26) with highest adhesion and three isolates (P7, P23, P42) with lowest adhesion are labelled with numerical values.

In silico and in vitro studies, as well as animal models of infection, have shown the pathogenic potential of foodborne ExPEC to humans [68]. ExPEC can efficiently colonize the human GI tract, potentially contributing to their long-term carriage, whereby they can opportunistically infect other body sites, leading to extraintestinal infections [69], as reviewed in [70]. Therefore, the ability of the 43 poultry ExPEC to attach to Caco-2 cells as a model of infectivity was evaluated relative to a highly adherent *E. coli* O157:H7 strain. Most of the poultry isolates (27/43) clustered within a ± 0.3 log CFU/mL range, indicating that they had adhesion efficiencies similar to the *E. coli* O157:H7 control (Figure 2). Among these, 22 isolates were ABR, 3 formed moderate-to-high biofilm mass, and 1 was intermediately resistant to QAC. Heterogeneity within the set of poultry ExPEC was observed. Five isolates [P12 (+0.94), P27 (+0.61), P26 (+0.58), P35 (+0.53), and P39 (+0.31)] had higher adhesion abilities than *E. coli* O157:H7. Eleven isolates showed significantly (*p* < 0.05) lower adhesion with strongest reductions observed for four isolates (P7 (−1.17), P23 (−1.06), P34 (−0.86), and P42 (−0.86) (Figure 1)). Adhesion by many (62.8%) of the poultry ExPEC to intestinal epithelial cells suggests their ability to colonize the intestine and serve as a source for extraintestinal infections. Furthermore, isolate P27 displayed ABR and QAC resistance, emphasizing the increased risk some poultry ExPEC may pose to humans.

## 4. Conclusions

This study found that poultry can serve as a reservoir for antibiotic and sanitizer resistant ExPEC. Furthermore, ABR ExPEC from poultry are capable of adhering to human GI tract cells, which can then serve as a source for ExPEC infections, including drug-resistant infections leading to treatment failure. Evaluation of these isolates for their ability to infect extraintestinal sites and comprehensive genome characterization is needed for better understanding of their potential impact on human health.

## Figures and Tables

**Table 1:** Characteristics of ExPEC isolates from human clinical and poultry meat samples.

Isolate ^a^	Source ^b^	Geographical Location ^c^	Reference ^d^	Antibiotic Resistance ^e^	Sanitizer Resistance		Biofilm Score ^g^
					Untreated	300 ppm QAC ^f^	
H1	UTI	MW, USA	This study	ABR	8.98 ± 0.04	ND	1
H2	UTI	PW, USA	This study	S	8.70 ± 0.02	ND	1
H3	UTI	SE USA	This study	MDR	8.90 ± 0.01	ND	1
H4	UTI	SE, USA	This study	MDR	8.91 ± 0.08	4.44 ± 2.11	0
H5	UTI	MW, USA	This study	MDR	8.92 ± 0.07	2.98 ± 1.14	0
H6	UTI	NE, USA	This study	S	8.72 ± 0.06	ND	1
H7	UTI	NE, USA	This study	ABR	8.77 ± 0.09	ND	0
H8	UTI	NE, USA	This study	S	8.72 ± 0.06	4.09 ± 1.06	1
H9	UTI	NE, USA	This study	S	8.83 ± 0.05	ND	0
H10	UTI	NE, USA	This study	ABR	8.81 ± 0.02	ND	1
H11	UTI	NE, USA	This study	S	8.83 ± 0.08	ND	1
H12	UTI	NE, USA	This study	S	8.85 ± 0.02	ND	1
H13	UTI	NE, USA	This study	MDR	8.90 ± 0.02	4.09 ± 0.68 ^I^	1
H14	UTI	NE, USA	This study	S	8.86 ± 0.08	ND	0
H15	UTI	NE, USA	This study	S	8.81 ± 0.07	ND	0
H16	UTI	NE, USA	This study	S	8.78 ± 0.09	3.78 ± 0.88	1
H17	UTI	PA, USA	This study	S	8.93 ± 0.05	6.72 ± 0.19 ^R^	1
H18	UTI	PA, USA	This study	S	8.88 ± 0.05	ND	1
H19	UTI	NE, USA	This study	ABR	8.77 ± 0.02	ND	0
H20	UTI	NE, USA	This study	S	8.83 ± 0.04	ND	0
H21		NA	NA	MDR	8.78 ± 0.05	ND	1
H22		NA	NA	MDR	8.87 ± 0.10	ND	0
H23		NA	NA	MDR	8.73 ± 0.07	ND	1
H24		NA	NA	MDR	8.84 ± 0.05	ND	1
H25		NA	NA	MDR	8.82 ± 0.10	ND	0
H26	UTI	NA	[43]	S	8.96 ± 0.09	3.41 ± 0.36	1
H27	UTI	PA, USA	[44]	ABR	8.88 ± 0.11	ND	1
H28	UTI	PA, USA	[45]	ABR	8.90 ± 0.01	ND	0
H29	UTI	PA, USA	[45]	ABR	8.87 ± 0.09	ND	0
H30	UTI	NA	[45]	S	8.73 ± 0.06	ND	0
H31	NM	NA	[46]	S	8.86 ± 0.03	ND	1
H32	UTI	PW, USA	[47]	S	8.90 ± 0.03	ND	2
H33	UTI	NE, USA	[48]	S	8.92 ± 0.03	ND	0
H34	UTI	NA	This study	ABR	8.83 ± 0.05	2.45 ± 0.78	0
H35	UTI	NA	This study	S	8.59 ± 0.07	3.26 ± 0.59	1
H36	UTI	MW, USA	[49]	MDR	8.99 ± 0.05	ND	0
H37	NM	Netherlands	[50]	S	8.90 ± 0.05	ND	1
H38	NM	Netherlands	[50]	MDR	8.90 ± 0.00	ND	1
H39	NM	Netherlands	[50]	S	8.88 ± 0.03	ND	1
H40	NM	Netherlands	[50]	S	8.88 ± 0.04	0.09 ± 0.12	1
P1	Chicken	Mexico	This study	MDR	8.67 ± 0.20	ND	0
P2	Chicken	Mexico	This study	MDR	8.83 ± 0.10	ND	0
P3	Chicken	Mexico	This study	MDR	8.63 ± 0.17	ND	2
P4	Chicken	Mexico	This study	MDR	8.85 ± 0.11	ND	1
P5	Chicken	Mexico	This study	MDR	8.90 ± 0.16	ND	3
P6	Chicken	Mexico	This study	MDR	8.76 ± 0.12	ND	1
P7	Chicken	Mexico	This study	MDR	8.42 ± 0.25	ND	0
P8	Chicken	Mexico	This study	MDR	8.52 ± 0.23	ND	1
P9	Chicken	Mexico	This study	MDR	8.56 ± 0.29	ND	1
P10	Chicken	Mexico	This study	MDR	8.66 ± 0.20	ND	2
P11	Chicken	Mexico	This study	MDR	8.90 ± 0.09	ND	1
P12	Turkey	PW, USA	This study	S	8.80 ± 0.10	3.57 ± 0.46	1
P13	Chicken	MW, USA	This study	ABR	8.63 ± 0.16	ND	0
P14	Chicken	PW, USA	This study	ABR	8.69 ± 0.19	ND	1
P15	Chicken	MW, USA	This study	S	8.71 ± 0.13	ND	0
P16	Chicken	SE, USA	This study	S	8.71 ± 0.23	ND	1
P17	Chicken	PA, USA	This study	MDR	8.71 ± 0.17	ND	0
P18	Chicken	PW, USA	This study	ABR	8.70 ± 0.21	ND	0
P19	Chicken	NE, USA	This study	ABR	8.76 ± 0.15	ND	0
P21	Chicken	NE, USA	This study	S	8.82 ± 0.17	ND	0
P22	Chicken	NE, USA	This study	ABR	8.43 ± 0.20	ND	1
P23	Chicken	NE, USA	This study	S	8.77 ± 0.19	ND	0
P24	Chicken	PA, USA	This study	S	8.82 ± 0.14	ND	1
P25	Chicken	SE, USA	This study	ABR	8.84 ± 0.15	4.72 ± 0.71	0
P26	Chicken	NE, USA	This study	S	8.59 ± 0.17	ND	1
P27	Chicken	SE, USA	This study	ABR	8.78 ± 0.16	5.4 ± 0.8 ^I^	1
P28	Chicken	SE, USA	This study	S	8.78 ± 0.15	ND	1
P29	Chicken	MW, USA	This study	ABR	8.86 ± 0.10	ND	0
P30	Chicken	SE, USA	This study	MDR	8.83 ± 0.14	ND	2
P31	Chicken	NE, USA	This study	S	8.86 ± 0.14	ND	0
P32	Chicken	NE, USA	This study	S	8.78 ± 0.18	ND	0
P33	Turkey	SE, USA	This study	ABR	8.84 ± 0.39	ND	1
P34	Turkey	SE, USA	This study	MDR	8.72 ± 0.18	ND	0
P35	Turkey	MW, USA	This study	S	8.85 ± 0.15	ND	0
P36	Chicken	NE, USA	[19]	MDR	8.78 ± 0.10	ND	1
P37	Chicken	NE, USA	[19]	ABR	8.79 ± 0.05	ND	1
P38	Chicken	NE, USA	[19]	ABR	8.73 ± 0.11	ND	0
P39	Chicken	NE, USA	[19]	S	8.80 ± 0.07	ND	0
P40	Chicken	NE, USA	[19]	ABR	8.82 ± 0.05	ND	1
P41	Chicken	NE, USA	[19]	ABR	8.79 ± 0.01	3.17 ± 0.29	1
P42	Chicken	NE, USA	[19]	MDR	8.86 ± 0.08	ND	1
P43	Chicken	NE, USA	[19]	ABR	8.82 ± 0.06	ND	1
P44	Chicken	SE, USA	[19]	S	8.77 ± 0.18	ND	1
BHI		-	-	-	-	-	0
43894 OR 22/30	Human O157:H7		[35]	-	-	-	3

^a^, H: human, P: poultry; ^b^, UTI: Urinary tract infection; NM: neonatal meningitis; ^c^, NE: North East; SE: Southeast; MW: Midwest; PW: Pacific West; PA: Plains; NA: data not available; ^d^, NA: not available; ^e^, S: antibiotic sensitive; ABR: antibiotic resistant; MDR: multidrug resistant; ^f^, ND: no detection; ^g^, 0: no biofilm; 1: mild biofilm; 2: moderate biofilm; 3: high biofilm. ^I^: intermediately resistant; ^R^, Resistant.

## Data Availability

The original contributions presented in this study are included in the article. Further inquiries can be directed to the corresponding author.
